# The Exponentially Weighted Moving Average Procedure for Detecting Changes in Intensive Longitudinal Data in Psychological Research in Real-Time: A Tutorial Showcasing Potential Applications

**DOI:** 10.1177/10731911221086985

**Published:** 2022-05-22

**Authors:** Arnout C. Smit, Evelien Schat, Eva Ceulemans

**Affiliations:** 1 University of Groningen, the Netherlands; 2 VU Amsterdam, the Netherlands; 3 KU Leuven, Belgium

**Keywords:** EWMA procedure, online monitoring, ecological momentary assessment, actigraphy, statistical process control

## Abstract

Affect, behavior, and severity of psychopathological symptoms do not remain static throughout the life of an individual, but rather they change over time. Since the rise of the smartphone, longitudinal data can be obtained at higher frequencies than ever before, providing new opportunities for investigating these person-specific changes in real-time. Since 2019, researchers have started using the exponentially weighted moving average (EWMA) procedure, as a statistically sound method to reach this goal. Real-time, person-specific change detection could allow (a) researchers to adapt assessment intensity and strategy when a change occurs to obtain the most useful data at the most useful time and (b) clinicians to provide care to patients during periods in which this is most needed. The current paper provides a tutorial on how to use the EWMA procedure in psychology, as well as demonstrates its added value in a range of potential applications.

It is increasingly recognized that human psychology is highly changeable and dynamic (i.e., constantly in motion): Affect, behavior, environmental factors, and severity of psychopathological symptoms do not remain static throughout the life of an individual, but rather they change over time (Diener & Emmons, 1984; Guastello et al., 2008; Mischel & Shoda, 1995; Reis et al., 1980). Intensive longitudinal data is needed to properly investigate these changes and shed light on the rules governing these changes (Hayes et al., 2007; Molenaar, 2004; Molenaar & Campbell, 2009; Nelson et al., 2017). Ecological Momentary Assessment (EMA) is a popular method to collect such data. This method involves sampling the affect, behavior, and or environmental factors of an individual, typically several times a day for multiple days (Larson & Csikszentmihalyi, 1983; Myin-Germeys et al., 2018, 2009; Shiffman et al., 2008; Stone & Shiffman, 1994). Since the rise of the smartphone, collection of such data has become far more feasible in psychological research, sparking a large increase in both the interest in, as well as the availability of such data. Studies collecting EMA data for 1 or 2 weeks can be used to investigate how dynamics differ between persons or how experiences and behaviors covary within persons (e.g., higher levels of negative affect in stressful situations). However, in recent years it has been shown that it is also feasible to collect EMA data over a longer period of time (Schreuder et al., 2020; Smit et al., 2019; Smit, Snippe, et al., n.d.; Wichers et al., 2016, 2020). Such an extended research period increases the likelihood of substantial within-person changes in affect, behavior, and/or severity of psychopathological symptoms occurring during the research period, and allows researchers to investigate these intra-individual changes (Mehl & Conner, 2012; Nelson et al., 2017; Smit, Snippe, et al., n.d.; Wright & Woods, 2020).

Unfortunately, many of the methods currently used to analyze EMA data are unfit for investigating within-person changes over time. For example, commonly used (partial) correlation analysis, and (vector) autoregressive (VAR) models require the data to be stationary (Lütkepohl, 2005). In an intuitive sense, stationarity means that the statistical properties of the process generating the time series (e.g., means, variances, and serial correlations) remain the same throughout the research period. The assumption of stationarity directly contradicts the goal of studying change over time, and therefore any model assuming stationarity cannot be used to investigate such changes. Of course, methods do exist that do not assume stationarity, and can be used for studying change over time in intensive longitudinal data. However, most methods that do not assume stationarity, such as time-varying (V)AR models (Bringmann et al., 2018), and change-point detection methods (Cabrieto et al., 2018, 2019), can only be applied after the data collection has been completed. This is unfortunate, as it could be useful to be able to detect within-person changes in intensive longitudinal data prospectively, in real-time.

First, being able to detect changes in real-time provides new research opportunities. In most studies, EMA questionnaires are kept short to keep participant burden manageable. When within-person changes in psychological functioning can be detected in real-time, additional questionnaires or qualitative interviews could be added at these highly relevant times. Since this additional information can be gathered when recall bias is still limited, such additional data could provide otherwise unobtainable insights in how and why changes occur. Second, detecting changes in real-time could be highly relevant for clinical applications. Interventions could be started or adapted as soon as a change is detected. In some cases, it may even be possible to detect changes when they are still relatively small and harmless, and start interventions before these small changes grow into more problematic ones (e.g., a depressive episode). Real-time change detection could be a big step toward providing the right intervention, to the right patient, at the right time.

Recently it was shown that it may be possible to detect intra-individual changes in intensive longitudinal psychological data in real-time using statistical process control (SPC) methods (Schat et al., 2021; Smit et al., 2019; Smit & Snippe, n.d.). SPC methods were originally developed to monitor an industrial production process over time and indicate when changes in the process occurred. Several univariate SPC methods exist, including the Shewhart procedure (Shewhart, 1931), cumulative sum procedure (Page, 1954), and the exponentially weighted moving average (EWMA) procedure (Roberts, 1959). In this paper, we will focus on the latter procedure. The EWMA procedure, which is applied to monitor the average level of a variable in real-time seems particularly useful, since it is relatively easy to implement and interpret. Moreover, all statistical process control procedures are based on some (potentially unrealistic) assumptions (e.g., normal distribution, independence of observations), but EWMA tends to be quite robust against violations of these assumptions. Finally, as the EWMA procedure has been applied in many fields and investigated for the better part of a century, its behavior and statistical properties are well understood. Though its behavior on simulated EMA data has been investigated recently (Schat et al., 2021) showing promising results, tutorial applications on different types of empirical intensive longitudinal data are needed to gain insight in the practical possibilities and limitations of this method in psychology research. The current paper aims to do this, by analyzing three different data sets. In addition, we provide a tutorial, R-code and practical recommendations, paving the way for future applications of the EWMA procedure in psychological research.

## Statistical Process Control

### General Idea

In this section we will introduce SPC using two examples. A first example stays close to the origins of SPC and stems from industry, in that we monitor the industrial process of filling water bottles. The output of the filling machine is tracked, where the scores we observe are the amount of ml in each bottle. Second, we consider an example from psychology, where we monitor the affective fluctuations of an individual as measured through EMA.

SPC procedures are based on the idea that even if a process remains the same, observations of that process will exhibit natural variability (in the statistical sense). For instance, there might be a small variation in the amount of ml in the water bottles, but overall the machine still functions well. Similarly, a person’s affect is expected to fluctuate over time, for instance due to contextual changes (Kuppens & Verduyn, 2017). If this natural variability of the observations is known, control limits can be set so that the vast majority of new observations of the machine or person fall between these control limits, as long as the process does not change (i.e., remains in-control). However, when the process characteristics do alter, features of the observations also change, which should result in more observations exceeding the control limits. For example, the filling machine can break down causing the produced water bottles to be empty. The monitored scores (i.e., amount of ml) will then no longer fall within in the in-control distribution/range, and the process should be flagged as out-of-control. For our second example, the monitored person may fall into a depression. Among others, this change will be reflected in an increase of negative affect (Clark & Watson, 1991; Watson et al., 1988). To summarize, exceeding the control limits indicates that it is likely that the data generating process has changed. The process is then considered to be out-of-control, and an intervention may be necessary. A mechanic may have to repair our bottle filling machine, whereas a therapist may have to check on our monitored person. On the contrary, if no out-of-control scores occur, there is no evidence that the process has changed, implying that intervening is not needed.

SPC procedures thus require two distinct research phases. In Phase I, the natural variability of the in-control data is captured and used to establish the in-control distribution. The estimated mean ˆµ_1_ and standard deviation ${\hat{\sigma }}_{1}$ of the Phase I data, are used to determine the upper control limit (UCL) and lower control limit (LCL). In Phase II, the actual monitoring starts and incoming data are compared with the in-control distribution, to determine whether and when the process goes out-of-control. The monitoring is commonly visualized in a control chart, where process scores are plotted against time.

### EWMA Procedure

The EWMA procedure (Roberts, 1959) was proposed to detect mean changes across time. It monitors a real-time running estimate of the average in a control chart rather than the original observations. Specifically, the procedure combines past information with current information and tracks a weighted sum of the original observations, where more recent observations receive higher weights. At each measurement occasion i ( i = 1, . . ., t ), the exponentially weighted moving average $z_{i}$ is calculated as:

$$z_{i}=\lambda x_{i}+\left(1-\lambda \right)z_{i-1}.$$

**Figure 1.**
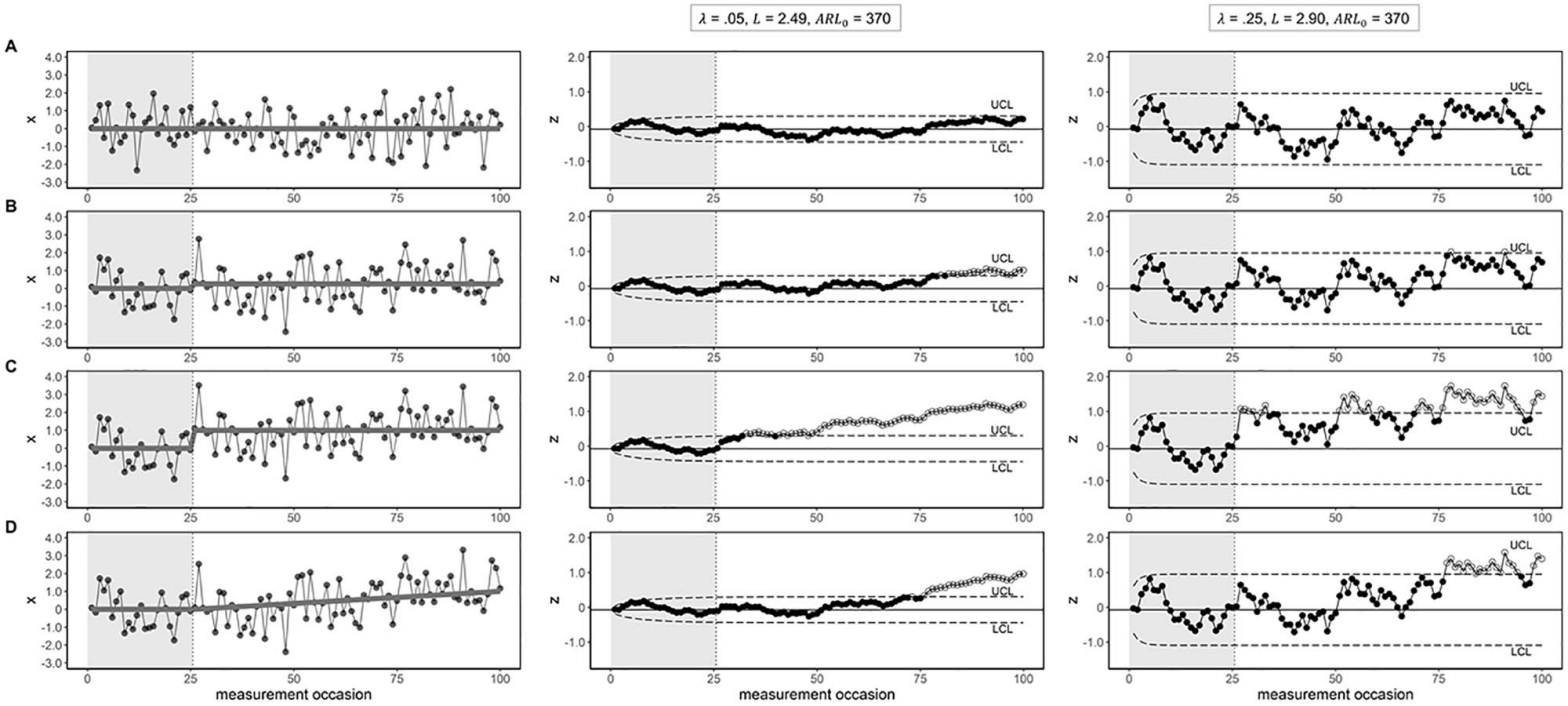
EWMA Control Charts for Different Sizes and Types of Mean Change. *Note.* The in-control Phase I data is indicated by the gray background shading; unshaded areas show Phase II data. The first column shows the simulated trends, indicated by the gray line. The raw data (indicated by black dots) was formed by adding identical noise (sampled independently from a normal distribution with µ_1_ ${\;}_{1}$ = 0 and $\sigma_{1}$ = 1) to each of these trends. As the raw data is identical except for the differences in trend, all control chart differences between 1a, b, c, and d are caused by the differences in the trends. The second and third columns show the EWMA control charts with different parameters. The dashed horizontal lines indicate the UCL and LCL. The solid horizontal line denotes the center line. The white dots indicate the out-of-control scores that fall beyond the control limits. (A) Data with no mean change. (B) Data with an abrupt change of .25 σ . (C) Data with an abrupt change of 1 σ . (D) Data with a gradual change (up to 1 σ ).

$x_{i}$ denotes the observation at measurement occasion i and the starting value $z_{0}$ is equal to the Phase I average ˆµ_1_. The parameter 0 <λ≤ 1 is the weight given to the current observation and thus also determines the rate at which the weights of the past observations decrease. In SPC literature, values between .05 and .25 are recommended, where lower values for λ are used to detect smaller mean changes (Montgomery, 2009). Figure 1 shows EWMA charts with λ = .05 (second column) and λ = .25 (third column), for different sizes (i.e., no, .25 σ and 1 σ change), and types (i.e., abrupt, gradual) of mean change. The first column displays the original observations. Phase I consists of 25 observations and Phase II consists of the remaining 75 observations. In Figure 1 we see that smaller λ values yield more smooth EWMA scores, as more weight is given to the previous observations. Despite the usual SPC recommendations, the appropriate value for λ may vary depending on the application, and new guidelines may be more appropriate for applications on EMA data. First simulation results suggest that λ values between .05 and .10 work well when using day averages (Schat et al., 2021).

Given λ , the Phase I average ˆµ1 and standard deviation ${\hat{\sigma }}_{1}$ , the upper and lower control limits are computed as:

$$UCL={\hat{\mu }}_{1}+L{\hat{\sigma }}_{1}\sqrt{\frac{\lambda }{\left(2-\lambda \right)}\left[1-{\left(1-\lambda \right)}^{2i}\right]}$$

and

$$LCL={\hat{\mu }}_{1}-L{\hat{\sigma }}_{1}\sqrt{\frac{\lambda }{\left(2-\lambda \right)}\left[1-{\left(1-\lambda \right)}^{2i}\right]}.$$

As i increases, the term $\left[1-{\left(1-\lambda \right)}^{2i}\right]$ approaches one, resulting in steady-state control limits. The parameter L determines the width of the control limits. In the next section, we will explain how to choose this L parameter in relation to a desired SPC performance.

Figure 1A shows a situation in which no mean change was introduced in Phase II, and the EWMA scores correctly remain within the control limits. An abrupt change of .25 σ and 1 σ are shown in Figure 1B and C, respectively. As expected, the first out-of-control score, indicated by the white dots, occurs earlier for the larger mean change. Although the SPC literature usually assumes abrupt changes, in psychology some changes may be gradual rather than sudden. In gradual changes, it is usually not possible to detect the beginning of the change, as the new process is still very similar to the in-control process. However, if a gradual change is large enough and/or continues for long enough, it can still be detected (see Figure 1D). The R code for generating the data and EWMA charts can be found at [https://osf.io/nf7zk/].

The EWMA procedure is often compared to other methods, such as the Shewhart and CUSUM procedures, as well as the simple moving average (SMA). Simulation studies by Schat et al. (2021) showed that the EWMA procedure performed considerably better than the Shewhart procedure. The CUSUM procedure, on the other hand, did not consistently perform worse than the EWMA procedure, but it is harder to implement as there is no simple formula to calculate the control limits. The SMA is often considered to be less complex than EWMA. Here, a time window of size k is slid along the time series, and in each window, the unweighted mean of k observations is computed. However, EWMA has several advantages over the SMA. First, EWMA is generally better at detecting small changes (Carson & Yeh, 2008; Montgomery, 2009; Roberts, 1959). Second, in some cases EWMA requires less observations than SMA to be effective (Roberts, 1959). Finally, the SMA cannot be obtained for the first few and last few observations (Carson & Yeh, 2008).

### ARL

The expected behavior of SPC procedures is usually expressed in term of the run length, which indicates at which Phase II observation the process goes out-of-control for the first time. In case the process remains in-control, an out-of-control EWMA would be a false positive (i.e., type 1 error). The expected run length until the first false positive is encountered is called the $ARL_{0}$ , and should preferably be as high as possible. On the contrary, in case the process does experience a change, the expected run length between the moment of the change until the first true positive is encountered is called the $ARL_{1}$ , and should ideally be as short as possible indicating high power. For more details on the run length distribution, we refer the reader to Schat et al. (2021). In the EWMA procedure, the L value in the computation of the control limits is related to the $ARL_{0}$ and $ARL_{1}$ values. Specifically, for a fixed λ a higher L leads to both a higher expected $ARL_{0}$ and a higher expected $ARL_{1}$ .

**Figure 2.**
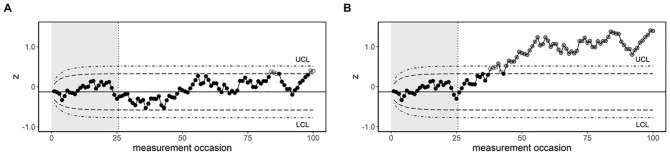
EWMA Control Charts Demonstrating the Impact of the Choice of $ARL_{0}$ . *Note.* The in-control Phase I data (indicated by the gray background shading) were independently sampled from a normal distribution with µ_1_ = 0 and $\sigma_{1}$ = 1; λ = .10 in both graphs. (A) EWMA chart for a process that remains in-control. (B) EWMA chart for a process that goes out-of-control with a mean change of 1 σ . The long-dashed (inner) lines are the UCL and LCL corresponding to $ARL_{0}$ = 100 and the dot-dashed (outer) lines are the UCL and LCL corresponding to $ARL_{0}$ = 1,000. The white dots indicate out-of-control scores only for $ARL_{0}$ = 100. The gray dots indicate the out-of-control scores for both $ARL_{0}$ values.

In practice, we suggest researchers to decide on a suitable $ARL_{0}$ value, which can easily be used to determine the correct L parameter and therefore the width of the control limits (see R code for details; [https://osf.io/nf7zk/]). Choosing higher $ARL_{0}$ values will lead to more conservative charts, whereas choosing lower $ARL_{0}$ values will lead to more sensitive charts. The influence of the $ARL_{0}$ is illustrated in Figure 2, where the control limits are based on an $ARL_{0}$ of 100 or an $ARL_{0}$ of 1,000. Figure 2A shows a situation in which the process is in-control in Phase II. In this case, the process remains within the control limits for $ARL_{0}$ = 1,000, whereas there are several out-of-control scores for $ARL_{0}$ = 100 (indicated by the white dots). Thus, the lower $ARL_{0}$ leads to the detection of false positives, while this was not the case for the higher $ARL_{0}$ . Figure 2B shows a situation in which the process goes out-of-control at the beginning of Phase II, due to a mean change of 1 σ . The white dots indicate the out-of-control scores with an $ARL_{0}$ of 100, and the gray dots indicate the out-of-control scores for both $ARL_{0}$ values. In this example, the first out-of-control score occurs at observation 38 and 41 for an $ARL_{0}$ of 100 and 1,000, respectively. Thus, with a lower $ARL_{0}$ , the change is detected slightly earlier. For purpose of illustration, the difference in the values was taken much larger than usual, as otherwise the differences were too subtle to visualize. This does, however, indicate that small differences in $ARL_{0}$ often do not lead to big differences in performance.

The $ARL_{0}$ value needs to be chosen prior to monitoring. A historically common value for the $ARL_{0}$ is 370 (Montgomery, 2009), which in the current context would mean that one would expect roughly one false positive each year. However, higher or lower $ARL_{0}$ values may be improve the usefulness of the chart in some cases. Researchers should consider the cost of the intervention associated with an out-of-control score. For example, it may not be very invasive for a mechanic to check a machine, whereas it may be more costly for a therapist to check up on a potential patient. If the cost of intervention is low, having more false positives is not problematic, and a lower $ARL_{0}$ can be chosen to facilitate the timely detection of changes. However, if the cost of intervention is high, a higher $ARL_{0}$ can be chosen to limit the number of false positives and unnecessary interventions. In this case, it also becomes more difficult to detect changes however, such that it takes longer to detect an actual change. To summarize: A lower $ARL_{0}$ means more power; a higher $ARL_{0}$ means fewer false positives.

Notice that lower values of λ will result in more narrow control limits when applying the formulae provided in order to preserve the chosen $ARL_{0}$ . This is why in Figure 1 the plots with a lower λ (middle) have more narrow control limits than the plots with a higher λ (right), even though this does not have a massive impact on when the EWMA first goes out-of-control. To summarize: Changing λ will affect the smoothness of the chart and the width of the control limits, but the chosen $ARL_{0}$ remains unchanged.

### Assumptions of SPC Procedures

SPC procedures are based on the assumption that observations are independent and normally distributed. This assumption is often violated in psychological research: observations are serially dependent (i.e., autocorrelated) and skewed distributed. Advantage of the EWMA procedure is that it is quite robust against violations of the assumption of normality (Montgomery, 2009). To deal with autocorrelation, Schat et al. (2021) recommend to monitor day averages rather than individual observations, which reduces or even removes the autocorrelation. Modeling and removing autocorrelation before running the EWMA is also an option (Montgomery, 2009; Smit et al., 2019). Although this procedure is more complicated especially in case of potential missingness in the data, it does allow the user to evaluate the EWMA at every individual observation rather than just once per day. An additional consequence of using day averages, is an increase in effect size as more fluctuations are averaged out, increasing the power of SPC procedures in detecting small changes. Therefore, we will monitor day averages in the remainder of this paper.

Next to distributional characteristics of the variable under investigation, potential users should be aware that sufficient Phase I data is needed to obtain accurate estimates of in-control behavior. Due to insufficient Phase I data, the in-control distribution may be either too wide or too narrow (i.e., too large or too small ${\hat{\sigma }}_{1}$ ), resulting in suboptimal control limits. This in turn influences the performance of the EWMA procedure: with too wide control limits it becomes more difficult to detect changes and with too narrow control limits the $ARL_{0}$ value becomes too low. In general, the more in-control Phase I days and observations, the more accurate the EWMA results. Exactly how many Phase I days and observations are needed to obtain reliable estimates and thus sufficiently good EWMA performance, depends on multiple aspects, such as the size of the change, the variance of the observed scores within the days and the distribution of the observations, as shown by the simulations by Schat et al. (2021). However, even with relatively little Phase I data (i.e., 28 days with 5 observations per day) and thus perhaps with slightly suboptimal control limits, the EWMA procedure was shown to have good results by Smit and Snippe (n.d.) when applying it to empirical data.

## Demonstrating the EWMA Procedure in Three Different Applications

In this section we present three applications that illustrate different purposes for using the EWMA procedure. In each application, we also vary one EWMA setting (i.e., Phase I length, λ , and expected $ARL_{0}$ value) to examine potential influences on the EWMA results. Application 1 focuses on the most common aim of EWMA applications, namely to detect detrimental changes in a process as soon as possible after they happen in real-time. We consider an example using EMA data obtained during a period in which the participant experienced two adverse life events that may trigger changes in the EMA data. Both life events are used to demarcate the start of Phase II, to examine the influence of the Phase I length on the EWMA procedure.

In Application 2, we again consider an example using EMA data in which changes in psychopathology occur, however this time without any sudden external triggers. In this application, there may a period before the onset of core symptoms in which an increase of prodromal symptoms can already be detected using the EWMA procedure. This has huge potential, as in such cases it would be possible to start an intervention when symptoms are still relatively harmless and manageable, which in turn may prevent a full-blown episode. Moreover, we investigate potential influences of the λ parameter on the EWMA procedure.

In Application 3, we investigate whether changes can be detected in passively collected data using the EWMA procedure. As it is typically difficult to predict during what period a change is likely to occur, relatively long research periods are needed to capture the change of interest. While recent studies have shown that EMA questionnaire data (such as in Applications 1 and 2) can be collected during a continuous period of several months (Helmich et al., 2020; Schreuder et al., 2020; Smit et al., 2019; Smit, Snippe, et al., n.d.), such a design may not be feasible or ideal in all study populations or for all research questions. In some cases it may be more suitable to use measurements with a lower participant burden than high frequency questionnaires, such as passive measurements of physiology or actigraphy (Kunkels et al., 2021). It is therefore useful to also investigate if such, more passive, measurements also show meaningful changes that can be detected using the EWMA procedure. In Application 3, we also examine potential influences of the expected $ARL_{0}$ value on the EWMA procedure.

### Application 1: Detecting Change After it Occurred

#### Purpose

Application 1 focuses on detecting detrimental changes in a process as soon as possible after they happen in real-time. The timely real-time detection of elevations in psychopathological symptom levels could help start interventions as soon as possible. In Application 1, we test whether increases in feeling down and in experiencing craving to use drugs can be detected after adverse life events that are expected to potentially trigger these symptoms.

#### Data

We demonstrate the EWMA procedure on the data of a participant who was monitored using a maximum of 4 semi-random EMA observations daily, for a period of 114 days (yielding a total of ~400 EMA observations). This participant was diagnosed with major depressive disorder (MDD), remitted substance abuse (amphetamines), panic disorder with agoraphobia, and borderline personality disorder. During the research period two large external life events happened: The participant’s grandmother passed away on day 45 of the study, and the COVID-19 lockdown started on day 74 of the study. We expect that characteristics of the EMA observations may change as a result of these life events. We investigate changes in the items “to what extent do you feel down at this moment,” and “did you feel like using amphetamines since the previous beep” in particular, as these were the items with the strongest conceptual links to MDD and substance abuse, respectively. For a more complete description of this study, see Dejonckheere et al. (2021).

#### Results

**Figure 3:**
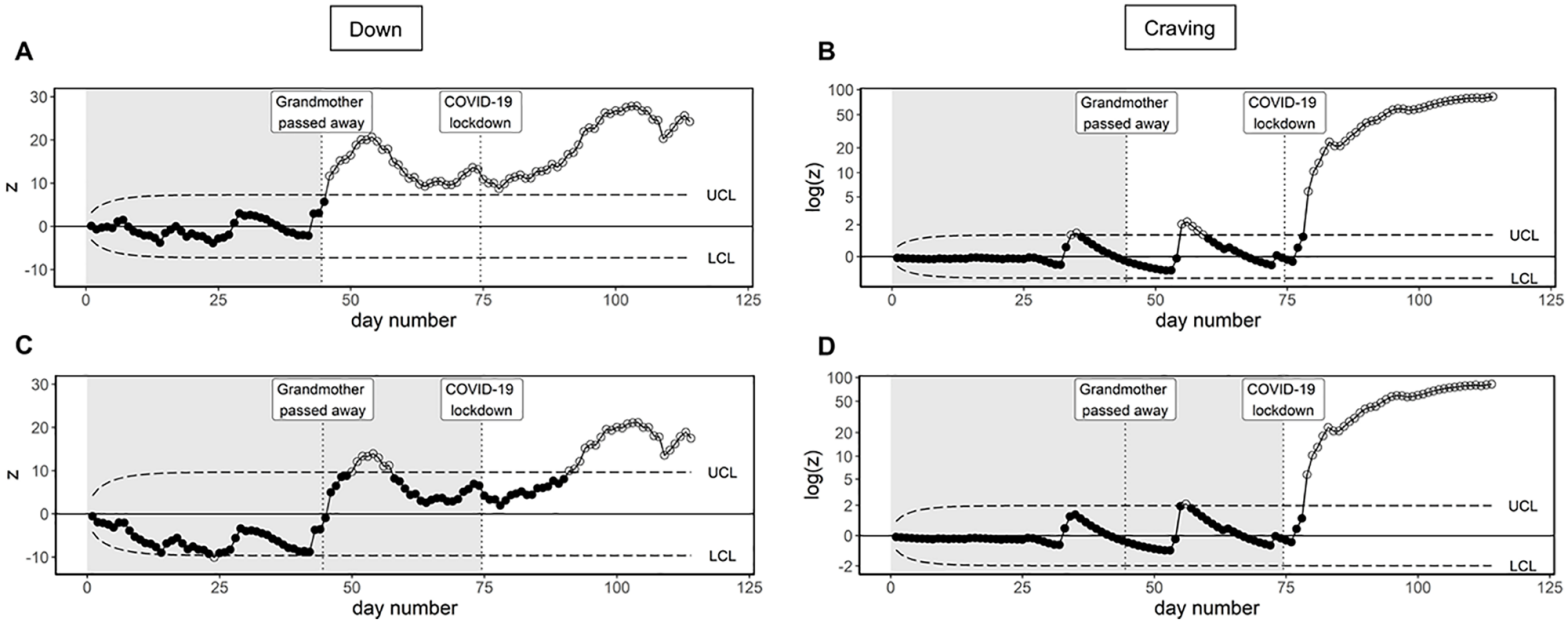
EWMA Control Charts With Varying Phase I Lengths. *Note.* Phase I lengths are indicated by the gray background shading. (A) EWMA chart of “down” with a “Phase I” period of 44 days. (B) EWMA chart of “craving” with a “Phase I” period of 44 days. (C) EWMA chart of “down” with a “Phase I” period of 74 days. (D) EWMA chart of “craving” with a Phase I period of 74 days.

Figure 3A and B shows EWMA control charts of the day averages of “down” and “craving,” respectively. The following settings were used: $ARL_{0}$ = 370, λ = .10, and a Phase I length of 44 days (i.e., all days before the participant’s grandmother passed away). The EWMA procedure detected a clear increase in “down,” shortly after the participant’s grandmother passed away. Although an increase in “down” can be viewed as a healthy reaction to the death of a close one, the EWMA procedure also shows that “down” does not return to its Phase I level and remains elevated for the remainder of the research period. The passing of the participant’s grandmother did not seem to increase “craving,” but shortly after the COVID-19 lockdown started, a clear increase in “craving” was found. This information could have been used in real-time to start an intervention and hopefully prevent the transition from elevated craving to the recurrence of substance abuse.

This example shows that different events may trigger changes in different symptoms. The passing of the participant’s grandmother may have led to a persistent change in “down,” which may be most relevant in the context of the major depressive disorder; the COVID-19 lockdown seems to have led to an increase in “craving,” which could be relevant in the context of the remitted substance abuse.

#### Impact of Chart Settings

This application provides an opportunity to gain insight in the relevance of the choice of the length of Phase I. As, to date, no EMA datasets have been gathered with the specific goal to analyze them using the EWMA procedure, the data used in Phase I has not specifically been collected with the aim of using it as in-control data. Therefore, the Phase I period needed to be defined post hoc in this case, with the possibility that a relevant change already occurred during Phase I.

Figure 3C and D shows EWMA control charts of “down” and “craving,” using the same EWMA settings, but setting the Phase I period to 74 days rather than 44 (i.e., all days before the COVID-19 lockdown). For “craving,” no change occurred during Phase I, and the different choice of Phase I period had minimal impact on the results. The change in “down” due to the passing of the participant’s grandmother now falls within the Phase I period. This had two effects on the chart: (1) the participant experienced a higher average level of “down” during Phase I, shifting the control limits upward and (2) the EMA observations of “down” had a higher variance during Phase I, widening the control limits. This meant that a larger upward change in “down” is needed in Phase II before the EWMA procedure marks it as significantly different from Phase I. Although in this specific case, an increase in “down” after both life events could still be detected, the EWMA goes back in-control between the two life events. This creates the impression that “down” returned to its normal level, even though it remains significantly higher than in the period before the participant’s grandmother passed away. When applied in a clinical setting, this may mean that with a 44-day Phase I an intervention would be started from the moment the participant’s grandmother passed away, while using a 74-day Phase I this intervention may have been stopped when the scores went in-control again.

### Application 2: Detecting Change Before it Occurs

#### Purpose

Even before the onset of core symptoms there may be a period in which early changes can already be detected. For the purpose of such early stage detection, focusing on items that are expected to increase during the prodromal phase of a disorder might be useful, rather than items that are the closest proxies for core symptoms. Based on this idea, it has been hypothesized that an increase in the item “I feel restless” may be found before the onset of core depressive symptoms (Smit et al., 2019; Smit & Snippe, n.d.), as symptoms of anxiety often precede depressive episodes (Hetrick et al., 2008; Pede et al., 2017). Notice that items such as “I feel down” may not yet show a clear mean change during the prodromal phase, but rather only increase once the core depressive symptoms start to increase.

In Application 2, EMA data was collected before (Phase I), during, and after (both Phase II) gradual discontinuation of antidepressant medication. We test whether an increase in restlessness can be detected *before* the start of core depressive symptoms, using the EWMA procedure. The R code to construct the EWMA control charts for Application 2 can be found at [https://osf.io/nf7zk/].

#### Data

The EWMA procedure was performed on the day averages of the publicly available data described in Wichers et al. (2016). One participant filled out a maximum of 10 EMA questionnaires daily before, during, and after gradual antidepressant discontinuation (tapering), yielding a total of 1,474 EMA observations over a continuous period of 239 days. From days 42 to day 98, double blind tapering of the participant’s antidepressant medication started. It was hypothesized that this change in context may lead to an increase in depressive symptoms, and around day 127 of the experiment, a sudden increase in depressive symptoms indeed occurred.^1^ For a more complete description of this study, see Wichers et al. (2016).

#### Results

**Figure 4.**
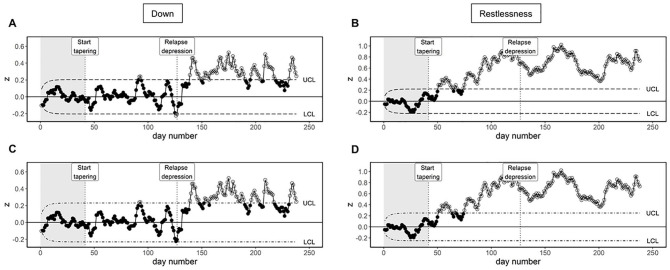
EWMA Control Charts Based on Varying $ARL_{0}$ Values. *Note.* The Phase I period of 41 days is indicated by the gray background shading, lasting until the start of tapering denoted by the first vertical line. The second vertical line denotes the depressive relapse around day 127. (A) EWMA chart of “down” with control limits based on an $ARL_{0}$ of 370. (B) EWMA chart of “restlessness” based on an $ARL_{0}$ of 370. (C) EWMA chart of “down” with control limits based on an $ARL_{0}$ of 1,000. (D) EWMA chart of “restlessness” based on an $ARL_{0}$ of 1,000.

Figure 4A and B shows EWMA control charts of “down” and “restlessness,” respectively. The following settings were used: $ARL_{0}$ = 370, λ = .10, and a Phase I period of 41 days (i.e., all days before tapering started). Although “down” exceeds the control limits briefly around day 92 and 126 of the study, it remains predominantly out-of-control after day 139 of the study (i.e., 12 days after the increase in depressive symptoms). “Restlessness” exceeds the upper control limit almost 3 months sooner, on day 51 of the study. Due to the real-time nature of the EWMA procedure, it may have been possible to put tapering on hold, return to a higher dosage of antidepressant medication, or start psychological treatment, long before the start of core depressive symptoms.

Note that the chart itself does not provide information on why “restlessness” starts changing at this early stage. The change may indicate a rise in prodromal symptoms as hypothesized, but could also reflect direct effects from antidepressant tapering on the EMA data. However, it can clearly be seen how relevant the choice of variable can be in the timely detection of changes using the EWMA procedure.

#### Impact of Chart Settings

This application provides an opportunity to gain insight in the relevance of the choice of the $ARL_{0}$ . The item “down” exceeded the upper control limits briefly around day 92, which can be seen as a significant change in “down” compared to Phase I. This may have allowed us to intervene before the increase in depressive symptoms (though still not as early as based on “restlessness”). However, based on visual inspection it does not seem that a clear change in “down” had already occurred before the depressive relapse around day 127 of the study. Combined with the fact that “down” also exceeded the lower control limit around day 126, we may expect that both brief out-of-control periods were actually false alarms. By increasing the $ARL_{0}$ , the type I error of the procedure can be decreased, at the cost of less power to detect changes.

Figure 4C and D shows the difference between the control chart using the commonly used $ARL_{0}$ = 370, and a much larger $ARL_{0}$ = 1,000 to provide sufficient contrast. It can be seen that the choice of $ARL_{0}$ determines how wide the control limits are, while the rest of the chart is not affected. Despite the large difference in the settings for the $ARL_{0}$ , the chart performance is quite robust against this change.

### Application 3: Passively Collected Data

#### Purpose

If changes in psychopathology can be detected using passively collected data, this could be an important step toward reducing the participant burden in research using the EWMA procedure. Theoretically, any time series that is hypothesized to change in a meaningful way compared with the Phase I period can be used to construct a control chart. For example, we may hypothesize that physical activity measured using actigraphy reduces when depressive symptoms increase, as physical activity tends to be lower in depressed patients (Burton et al., 2013). Although this link may not be as direct as the link between an individual’s mood and depression, intensive longitudinal data on physical activity has the advantage that it can be collected using accelerometers that require no active attention from participants. In Application 3 we apply the EWMA procedure to actigraphy data, and test if a reduction in physical activity can be detected in real-time before or shortly after an increase in depressive symptoms.

#### Data

For the actigraphy measurements, participants wore the MotionWatch 8 accelerometer by CamNtech (CamNtech, 2020; Kunkels et al., 2020) on their wrist for a continuous period of 4 months during and shortly after (gradual) antidepressant discontinuation. A pilot case with 177,120 1-minute bins of actigraphy data covering 123 days will be used for the current study. The participant experienced an increase in depressive symptoms around day 68 of the study period. For a more complete description of this study, see Smit et al. (n.d., 2020).

#### Results

**Figure 5.**
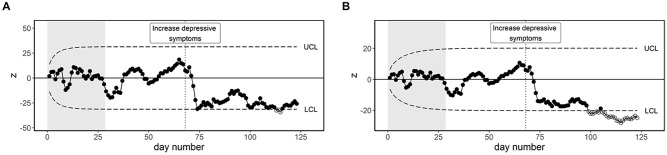
EWMA Control Charts of the Actigraphy Data With Varying λ Values. *Note.* The Phase I period of 28 days is indicated by the gray background shading. Increase in depressive symptoms is indicated by the vertical line on day 68. (A) EWMA chart with λ = .10. (B) EWMA chart with λ = .05.

Figure 5A shows EWMA control chart of day averages of actigraphy data. The following settings were used: $ARL_{0}$ = 370, λ = .10, and a Phase I period of 28 days (i.e., the same length of Phase I that was used in Smit & Snippe, n.d.). Before the increase in depressive symptoms, the EWMA of actigraphy data remains between the control limits, indicating the participant’s physical activity during this period was similar to the Phase I period. After the increase in depressive symptoms, a clear drop in physical activity can be seen, with the first out-of-control score on day 111, 43 days after the increase in depressive symptoms. This demonstrates that real-time changes in actigraphy data could be detected for this participant with the EWMA procedure, and may be indicative of the change in depressive symptoms the participant experienced.

#### Impact of Chart Settings

This application provides an opportunity to gain insight in the relevance of the choice of the λ parameter. Figure 5B shows the EWMA control chart of the actigraphy data using λ = .05 rather than λ = .10. As can be seen, the lower λ results in a somewhat smoother control chart, that changes more slowly over time. In general, lower values of λ are needed when the goal is to detect small changes, but using a lower λ , changes in the raw data do not affect the EWMA score as quickly, and may delay the detection of changes. In Figure 5B it can be seen that, though the chart looks different visually, the decrease in physical activity is still detected around the same time when λ = .05 (first out-of-control score on day 106). This shows that the performance of the procedure was not very sensitive to changes in the choice of λ .

### Summary of the EWMA Settings

Table 1 provides an overview of the EWMA settings discussed in the three applications (i.e., Phase I length, $ARL_{0}$ , λ ). It summarizes the effects on detecting mean changes as well as their impact on our application results.

**Table 1:** Overview of the EWMA Settings.

EWMA setting	Description	Effects on detecting mean changes
Phase I length	Amount of available in-control data, which is used to obtain estimates of the process’ in-control behavior (i.e., µˆ_1_ ${\hat{\mu }}_{1}$ and ${\hat{\sigma }}_{1}$ ). These estimates are used to calculate the control limits of the EWMA procedure.	Insufficient Phase I data may lead to an in-control distribution which is either too wide or too narrow (i.e., too large or too small ${\hat{\sigma }}_{1}$ ). This can lead to suboptimal control limits, which influence the performance of the EWMA procedure. If the control limits are too wide, it becomes difficult to detect changes, whereas if the control limits are too narrow, the $ARL_{0}$ value becomes too low. In Application 1, the choice of the Phase I period had an impact on the EWMA results. By lengthening the Phase I period, the natural variability of the process changed due to a known event (death of the grandmother), which impacted the width of the control limits.
$ARL_{0}$	Expected run length until the first false positive (i.e., out-of-control score) is encountered.	A lower $ARL_{0}$ means more power due to more narrow control limits; a higher $ARL_{0}$ means fewer false positives due to wider control limits. In Application 2, the EWMA appeared to be quite robust against the change in $ARL_{0}$ value (i.e., 370 to 1,000).
λ	Weight parameter given to the current observation, where 0 <λ≤ 1. The remaining weight (1 - λ ) determines the rate at which the weights of the past observations decrease in the EWMA scores.	Lower values for λ are useful for detecting smaller mean changes. In Application 3, the performance of EWMA was not very sensitive to changes in the choice of λ (i.e., .05 and .10).

*Note.* EWMA = exponentially weighted moving average.

## Discussion

The three applications in the current paper demonstrate the potential usefulness of the EWMA procedure in psychological research, and demonstrate that it is feasible to apply it on a range of relevant time series data. Furthermore, this study shows that it is possible to construct person specific control charts with individualized control limits, that allow us to monitor single individuals without the need to obtain a sample of similar participants. This means the EWMA procedure allows the user to personalize variables and parameters for each person individually in a relatively simple way. In addition, the EWMA procedure can be used to analyze streaming data in real-time. This combination makes this method ideal for *N* = 1 research, and has high potential for clinical applications.

The results were in line with the idea that (a) different environmental factors can impact observed variables in different ways and (b) different variables may start to change at different stages in the development of psychopathology. In Application 1, feelings of sadness seemed to be triggered by the passing away of the participant’s grandmother and the COVID-19 lockdown, while “craving” only seemed to be strongly affected by the latter. In Application 2, the participant showed a large increase in “restlessness” during the prodromal stage of depression, while a large increase in “down” was found after the participant had already experienced a depressive relapse. This underlines the importance of variable selection in the EWMA procedure. As the EWMA procedure only requires the data of a single participant, the user has the freedom to select the variables that are expected to be most relevant for the individual under investigation. In Application 3, actigraphy data was used for the EWMA procedure, demonstrating the potential range of data types in which this method could be applied. This provides a lot of potential for personalizing the EWMA procedure, though this personalization needs further investigation.

In each of the three applications in this paper, we varied one EWMA setting (i.e., Phase I period, $ARL_{0}$ , and λ ) to illustrate their influence on the obtained results. In Application 1, two different Phase I periods were considered: The passing of the participant’s grandmother and the COVID-19 lockdown. We observed that the choice of the Phase I period is important, since the natural variability of a process may change during Phase I due to an, in this case known, event (the death of the grandmother). This may obviously impact the control limits, as we observed for “down.” Screening the Phase I period for such changes and evaluating their potential effect on the control limits, could help improve the performance of the EWMA procedure. In Application 2, the $ARL_{0}$ value was varied. The EWMA control charts with an $ARL_{0}$ of 370 and 1,000 differed only slightly, indicating that this chart setting had a minimal impact on the results, within this $ARL_{0}$ range. However, the $ARL_{0}$ remains the most direct way of controlling the balance between limiting type I error and maximizing power. Finally, in Application 3, we considered EWMA control charts with a λ of .05 and .10. Setting λ to .05 resulted in a smoother, more slowly fluctuating chart. In general, a lower λ allows for the detection of smaller changes, as it averages over more data, increasing the power. A higher λ means that the EWMA scores are more affected by recent observations, which theoretically could allow for quicker detection of substantial changes after they happen (though in the current sample this was not found as the first out-of-control score was actually found a few days later when using the higher λ ). Overall EWMA seems relatively robust to the considered variations of the $ARL_{0}$ and of λ . More research, however, is needed to establish a range of chart settings that leads to adequate results in psychological research, as some rules of thumb from the SPC literature may not generalize to psychology applications. Moreover, as a good practice, researchers can check whether the results converge across different chart settings. If similar results are found for a range of different settings, one can be more confident about the detected out-of-control scores.

Next to the chart settings, the statistical properties of the selected variables can also influence the performance of the EWMA procedure in terms of type I error and power to detect changes. Specifically, data are assumed to be independent over time and normally distributed. With autocorrelated data, the control limits are suboptimal, influencing both the type I error and power (e.g., Alwan & Roberts, 1988; Harris & Ross, 1991). However, a practical way to deal with autocorrelation in the context of EMA research is to monitor day averages rather than individual observations, as this reduces or even removes the autocorrelation (Schat et al., 2021). The EWMA procedure is known to be quite robust against violations of the normality assumption, meaning that the EWMA procedure can be applied to monitor variables that are skewed distributed (Schat et al., 2021; also see “craving” in Application 1). When items refer to more extreme behaviors (e.g., self-harm) or experiences (e.g., suspiciousness), observations may not vary at all during Phase I (i.e., floor effect items). For such items, control charts cannot be obtained using standard software. However, the principle of the control chart still holds and can be used in practice by manually setting the control limits at for instance 0, implying that any indication of self-harm will be flagged as an out-of-control score.

For other data characteristics, more research is needed to establish their impact on the EWMA procedure. First, it is unclear how missing data patterns (e.g., missing not at random) impact performance. For example, compliance has been shown to depend on the time of day (Rintala et al., 2019). Second, as holds for other time series methods (Vogelsmeier et al., 2019, 2021), SPC procedures implicitly assume measurement invariance across time, implying that participants always interpret the momentary questions in the same way as well as consistently use the answering scales. Given that SPC requires assessing participants across long stretches of time, finding ways of reducing or compensating for missing measurement invariance may improve the performance of the EWMA procedure. Third, ESM data may contain trends, such as diurnal patterns or specific context effects. Such trends violate the underlying EWMA assumption that all Phase I data are sampled from one and the same distribution. One way to deal with this is to detrend the data before applying EWMA, for instance by means of a smoothing procedure (Adolf et al., 2022; Cleveland et al., 1993) or by fitting a tailored time series model (for an overview, see Ariens et al., 2020). An alternative is to use the moving centerline EWMA (Mastrangelo & Brown, 2000). Fourth, though the EWMA procedure is aimed at detecting changes in the mean level, other changes (e.g., variance) can also affect the probability with which the control limits are exceeded. For instance, in Application 2, an alternative explanation for the two out-of-control periods around days 92 and 126 could be that antidepressant discontinuation lead to an increase in the variance of “down.” This is in line with the hypothesis that instability increases prior to transitions in depressive symptoms (Smit, Helmich, et al., n.d.; Wichers et al., 2016, 2020). If during Phase II the variance increases compared to Phase I, the process would tend to show more out-of-control periods.

SPC methods such as the EWMA procedure can be applied in real-time in the sense that the analysis can incorporate each new observation as soon as it becomes available. However, successful real-time implementation of the EWMA procedure comes with additional requirements. First, the collected data needs to be available for analysis shortly after it is obtained, and the data needs to be analyzed directly after becoming available. Although this is not necessarily very challenging as (a) several apps (e.g., PETRA and m-Path; Bos et al., 2022; Mestdagh et al., 2022) already upload data in real-time, and (b) there are many examples of analyzing regularly incoming data using the EWMA procedure (see Montgomery, 2009 for an overview of historical applications), researchers still need to keep this in mind when aiming to base an intervention on the EWMA procedure. Finally, changes can only be detected *after* they have occurred, and no form of analysis can change this. Whether this is soon enough to be useful strongly depends on the application. While in some cases it may be valuable to react as soon as possible *after* a patient has relapsed into substance abuse or depression, this would no longer allow us to prevent these highly detrimental changes. Preventive action is only possible if a variable can be found that already changes *before* the detrimental change occurs. For example, a patient may show increased craving for drugs before actually remitting into substance abuse (see Application 1), or start showing signs of restlessness before relapsing to depression (see Application 2).

It is important to note that in none of the applications above, the data was gathered specifically to be analyzed using the EWMA procedure. This means that the Phase I periods were defined post hoc, while real-time applications would require the user to define the Phase I period by collecting data on a predefined number of days before entering Phase II. Ideally, the Phase I data should be representative of how Phase II data is expected to behave when no change occurs in the participant, and should contain enough observations to reliably estimate control limits (see Schat et al., 2021 for guidelines for choosing an appropriate number of days). If Phase I contains data that is abnormal for the participant, this would impact the calculation of the control limits and therefore the performance of the chart. For example, as demonstrated in Application 1, if a change already occurs during Phase I, this can have a substantial effect on the width of the control limits. Also, life events may lead to abnormal variation in Phase I, which may not be expected to repeat in Phase II. It is important to note that most abnormalities in Phase I will represent additional variance on top of the natural variance we aim to capture, causing the control limits to be too wide and the EWMA procedure being on the conservative side. Thus, the main risk of a suboptimal Phase I period will be missing changes in Phase II, and improving the Phase I data will mainly help increase the power for detecting small changes in Phase II. As Application 1 and Schat et al. (2021) both demonstrate the importance of the Phase I period, future studies aiming to use the EWMA procedure should plan on collecting Phase I data. In addition, researchers may consider evaluating Phase I data to uncover and potentially control for abnormal sources of variance before beginning Phase II monitoring. Though the few existing empirical studies applying the EWMA procedure on EMA data seem to suggest that important changes are often large enough to be detected, even without having a strongly controlled Phase I period at hand (Smit et al., 2019; Smit & Snippe, n.d.). Information on how to collect Phase I data and evaluate its quality is provided elsewhere (Montgomery, 2009), but future studies are still needed to refine these procedures for application in psychology and test their usefulness.

Important property of the EWMA procedure is that it is a general purpose method, making it applicable in many research fields. This is an advantage from a statistical perspective, in that the framework is thoroughly tested and validated, as well as relatively straightforward to implement in a wide range of datasets. Whereas we focused on psychopathology, statistical process control can also be generalized to other fields in psychology, to study for instance personality development, cognitive development (gains or losses), or sudden gains in therapy. As evidenced by applications to daily COVID-19 data (Perla et al., 2021) or by applications to weekly or monthly hospital data (Thor et al., 2007), the frequency of the observations (e.g., weekly, monthly) does not play an important role in such generalizations, as long as the total number of in-control observations is high enough to obtain reliable control limits. However, this general purpose character is a disadvantage when looking for mechanistic insight in the onset and further development of psychopathology. Indeed, in contrast to network methods (Borsboom & Cramer, 2013) or computational models of affective dynamics (Loossens et al., 2020), SPC does not provide a causal theory about the etiology of psychopathology, such as vicious direct relations between symptoms. Also, though the EWMA procedure can be used to detect both sudden and gradual changes (see Figure 1), it does not provide information on whether the detected change occurred suddenly or gradually, and only provides an upper bound for the timing of the change. However, the simple interpretation of control charts may open new avenues of research regarding *how* and *why* changes occur. Specifically, both quantitative and qualitative measures could be intensified in out-of-control periods, to increase the information on *how* and *why* changes occurred.

Although the current paper showed the EWMA procedure in a range of *N* = 1 studies, applying this method in a sample of multiple participants that are all followed for an extended period using intensive longitudinal data could also be useful. This kind of research can be used to gain insight in how to personalize variables and parameters effectively, and investigate how the EWMA procedure will function when structurally applied in a specific population. Recently, Smit and Snippe (n.d.) performed such a study, where the *N* = 1 study in Application 2 was extended to a sample of 41 individuals. A pilot (Smit et al., 2019) was used to plan the study, and choose appropriate variables and settings for the EWMA procedure. The advantage of such a design is that it combines the personalized control limits for detecting within-person change, with the possibility to provide important between-persons summary statistics such as the sensitivity and specificity of the method, and the average timing of the first out-of-control EWMA score. Although a substantial investment of time and resources is required to obtain the data necessary, such studies do provide important information on the reliability, and overall usefulness of the EWMA procedure in psychological research and clinical practice.

Although SPC provides a practical statistical way of detecting significant changes in time series data, future research is needed to investigate the effectiveness of SPC-based interventions. Depending on the application and the intervention costs, benefits, and risks for both researchers/clinicians and participants/patients, parameters of the control chart need to be chosen in such a way that an appropriate balance between sensitivity and specificity is achieved. For low-cost interventions like Just-In-Time Adaptive Interventions, one could prefer a lower $ARL_{0}$ as the usefulness of quick detection may outweigh the issues that could arise from an increase in false positives; for more costly or burdensome interventions, such as restarting therapy or medication, a higher $ARL_{0}$ may be more appropriate, to avoid applying such an interventions in patients that do not need it.

In conclusion, the EWMA procedure is a general purpose statistical method that can be used to detect changes (a) in individual patients (i.e., without the need for a sample of multiple participants), allowing the user to personalize which variables are most relevant for each individual and (b) in real-time (i.e., data can be analyzed as soon as it is collected), making the EWMA is a unique new tool for analyzing time series data in psychology, that may be promising for clinical applications. Although some recent studies applying the EWMA procedure in multiple participants seem to confirm this potential usefulness (Smit et al., 2019; Smit & Snippe, n.d.), more research is needed to test the usefulness of this procedure in a wider range of psychological applications. The current study was an important step in this direction, by (a) demonstrating how the EWMA procedure was relatively straightforward to implement in three different psychological time series, and (b) exploring how the results could be used and interpreted in a range of applications.

## Notes

1 Depressive symptoms were measured weekly using the Symptom Checklist-90 (Arrindell & Ettema, 2003). A strong increase in depressive symptoms was detected in the data using the ECP package for detecting change points (James & Matteson, 2015). The clinical relevance of this change was confirmed by the participant and his psychiatrist decided to restart antidepressant medication shortly after the data collection was completed.
